# Mediating role of exercise behavioral intention in the relationship between exercise self-efficacy and physical activity levels in vocational education students

**DOI:** 10.3389/fpubh.2026.1833167

**Published:** 2026-06-18

**Authors:** Ziyang Jing, Rongxuan Zhai, Zihan Xu, Xudong Wu, Lejun Wang

**Affiliations:** 1 Shanghai Technician School, Shanghai University of Engineering Science, Shanghai, China; 2 Department of Sports Medicine, Sport and Health Research Center, Shanghai YangZhi Rehabilitation Hospital (Shanghai Sunshine Rehabilitation Center), Physical Education Department, Tongji University, Shanghai, China; 3 School of Public Administration, Hangzhou Normal University, Hangzhou, China

**Keywords:** exercise behavioral intention, exercise self-efficacy, mediating effect, physical activity, vocational education students

## Abstract

**Background:**

Social Cognitive Theory and the Theory of Planned Behavior suggest that self-efficacy influences physical activity through behavioral intention; however, evidence in vocational students is scarce.

**Objective:**

This study examined whether exercise behavioral intention mediates the association between exercise self-efficacy and physical activity levels among vocational education students in Shanghai.

**Methods:**

A cross sectional survey was conducted with 992 students from four vocational colleges. Participants completed validated measures of exercise self-efficacy (nine items, 1–10 scale), exercise behavioral intention (five items, 5-point Likert scale), and physical activity (IPAQ-SF). Data were analyzed using SPSS 25.0 (IBM Corporation, Armonk, New York, USA) and AMOS 26.0 (IBM Corporation, Armonk, New York, USA), adjusting for gender, age, and education level.

**Results:**

Exercise self-efficacy was positively associated with exercise behavioral intention (*r* = 0.612, *p* < 0.001) and physical activity (*r* = 0.322, *p* < 0.001). Mediation analysis showed that behavioral intention partially mediated this relationship (indirect effect = 0.122, 95% CI [0.085, 0.163]), accounting for 45.19% of the total effect.

**Conclusion:**

Exercise self-efficacy is associated with physical activity both directly and indirectly via behavioral intention in vocational students. Interventions enhancing self-efficacy and behavioral intention may support higher activity levels, though longitudinal studies are needed to confirm directionality.

## 1 Introduction

Vocational education plays a vital role in developing skilled workers to support industrial growth (1). In Shanghai, a leader in vocational education reform, significant attention is being given to the physical and mental wellbeing of students in vocational institutions, which include Vocational high school, Junior college, Vocational University (2). At this critical stage of their physical and psychological development, students' physical activity levels not only affect their current health but also influence their future career adaptability and quality of life (3, 4). However, research shows that vocational students tend to have low physical activity levels and lack motivation for sports participation (5). Improving their engagement in physical exercise and establishing a strong foundation for physical fitness remains a significant challenge in vocational education (6).

Exercise self-efficacy, rooted in Bandura's self-efficacy theory, refers to an individual's belief in their ability to perform physical tasks and maintain exercise behaviors (7). However, prior research presents inconsistent findings regarding its mediating role. Many studies have conceptualized exercise self-efficacy as a mediator linking various antecedents, such as social support, to physical activity. In contrast, other studies propose that behavioral intention may act as a more proximal mediator in the self-efficacy to behavior relationship (8, 9). For example, Hong and Chung (10) demonstrated that, among university students, self-efficacy had stronger direct effects on behavioral intention and behavior than on attitude, whereas the attitude intention behavior link was only significant for those not habituated to physical exercise. However, evidence for vocational students, whose schedules and training constraints differ substantially from general university populations, is scarce. This gap underscores the need to examine the mediation role of behavioral intention in this specific population. Conversely, Hou et al. (11) suggested that self-efficacy moderates, rather than mediates, the intention to behavior gap. These inconsistencies underscore a theoretical gap: it remains unclear whether behavioral intention functions as a mediator or moderator in the self-efficacy → physical activity pathway, particularly in understudied populations such as vocational students. Exercise behavior intention constitutes an individual's subjective plan and willingness to engage in future physical activity, serving as a critical bridge connecting psychological cognition with actual exercise behavior (12). In this context, research by Zhou et al. (13) and Bao (14) confirms that exercise behavior intention plays a significant mediating role in the relationship between psychological factors and physical activity.

Physical activity levels among student populations serve as a critical indicator of their physical and mental wellbeing, with contemporary research characterizing physical activity through a multidimensional lens. Research by Xu et al. (15) in adolescent cohorts highlights a strong correlation between physical activity and self-efficacy, identifying self-efficacy as a pivotal mediator linking external support to physical activity engagement. Studies by Gao et al. (16) and Jiang et al. (17) further substantiate that physical activity not only directly enhances physical and psychological health but also indirectly improves academic engagement and fortifies psychological resilience through elevated self-efficacy. Gao et al. (18) evaluated the reliability and validity of several physical activity questionnaires, including the IPAQ-SF, among Chinese college students. Their findings indicated that the IPAQ-SF, along with other subjective instruments, provided limited but acceptable reliability and validity for measuring moderate vigorous physical activity in this population, offering empirical support for its cautious use in research.

While these relationships have been extensively studied in general university students, research focusing on vocational education students remains limited. Vocational students differ from their academic track peers in several key ways that may alter the psychological pathways to physical activity. First, their academic schedules are heavily oriented toward professional skill development and mandatory internships, leaving limited discretionary time for structured exercise (19). Second, vocational students often exhibit a weaker foundation in physical activity and lower capacity for autonomous exercise planning, potentially increasing their reliance on conscious intentions to initiate activity (20). Third, recent evidence from Shanghai indicates a high prevalence of smartphone addiction (70.5%) and musculoskeletal disorders (38.0%) in this population (21), conditions that may directly reduce self-efficacy and impede the translation of intentions into action. These characteristics suggest that behavioral intention may serve as a particularly important, yet potentially vulnerable, mediator between self-efficacy and physical activity in vocational students compared with general university students. Therefore, studying this population is not merely an extension of previous research but provides a critical test of the generalizability of the mediation model linking self-efficacy, behavioral intention, and physical activity.

Despite this, most existing research focuses on general university students, highlighting the need for more targeted studies on the physical activity behaviors of vocational students. The proposed mediation model is grounded in the integration of Bandura's Social Cognitive Theory and Ajzen's Theory of Planned Behavior (TPB) (22, 23). According to Bandura, self-efficacy beliefs influence behavior both directly and indirectly by shaping individuals' goals, intentions, and expectations of outcomes (24, 25). Within the TPB framework, behavioral intention is the most immediate predictor of actual behavior, reflecting the motivational factors that drive action (23). In this context, self-efficacy is a crucial element of perceived behavioral control, enhancing an individual's confidence in their ability to perform the desired behavior. Empirical studies provide robust support for the mediating role of behavioral intention in the pathway from self-efficacy to physical activity. For instance, Lippke et al. (26) employed structural equation modeling to demonstrate that intention significantly predicts behavior through the mediation of action planning, and the strength of this mediation is moderated by individuals' self-efficacy levels. Similarly, Spink and Nickel (27) conducted a prospective study showing that self-regulatory self-efficacy partially mediates the relationship between attributional factors and exercise intention, providing longitudinal evidence for the causal chain from self-efficacy to intention. These findings align with the Social Cognitive Theory and the Theory of Planned Behavior, reinforcing the notion that self-efficacy enhances exercise intentions, which in turn translate into actual physical activity. By combining these theoretical and empirical insights, a clear pathway emerges in which exercise self-efficacy strengthens exercise behavioral intentions, which then lead to higher levels of physical activity. This pathway provides a solid theoretical and empirical basis for exploring the mediating role of exercise behavioral intention in vocational education students. Examining this integrated model in the understudied context of vocational students will provide evidence on whether the pathway generalizes across different educational settings.

## 2 Methods

### 2.1 Participants

A stratified random sampling approach was used to ensure representativeness of vocational education students in Shanghai. Three administrative districts (Pudong, Yangpu, and Minhang) were purposively selected to reflect urban, suburban, and peri urban characteristics. Within each district, one vocational high school, one junior college, and one vocational university were randomly chosen from the official roster of vocational institutions. At each selected school, two grades were randomly selected, and within each grade, three classes were randomly sampled. All students in the selected classes were invited to participate.

Questionnaires were distributed and collected during off class hours. Before administration, the study purpose and significance were explained to participants, and written informed consent was obtained from all students. A total of 1,005 questionnaires were distributed, of which 992 were valid, resulting in an effective response rate of 98.7%. The final sample included 324 vocational high school students, 342 junior college students, and 326 vocational university students, covering the three main levels of vocational education in Shanghai.

### 2.2 Methodology

#### 2.2.1 Exercise self-efficacy

The Exercise Self-Efficacy Scale, developed by Resnick and Jenkins (28) and translated by Lee et al. (29) was employed. This scale comprises nine items, each rated on a 1 to 10 point scale, with 1 indicating “impossible” and 10 indicating “very likely.” Item scores are summed, and higher total scores correspond to greater exercise self-efficacy. In this study, the scale exhibited a Cronbach's α coefficient of 0.827 and KMO was 0.96. The construct validity of the three factor measurement model (exercise self-efficacy, behavioral intention, physical activity) was assessed using confirmatory factor analysis (CFA) on the current sample. As reported in Section 3.1, the hypothesized three factor model fit the data acceptably [$\chi_{\left(116\right)}^{2}$ = 412.3, CFI = 0.951, TLI = 0.943, RMSEA = 0.056, SRMR = 0.045], supporting the factorial validity of the scales.

#### 2.2.2 Exercise behavior intention

The Exercise Behavior Intention scale was adapted from the instrument developed by Hong and Chung (10), which itself was constructed based on the Theory of Planned Behavior. The original scale comprises five items designed to assess an individual's subjective willingness and action plans for participating in physical exercise. Its reliability and validity have been previously validated within a university student population, demonstrating an internal consistency (Cronbach's α) of 0.84 and a clear unidimensional structure via exploratory factor analysis. In this study, the scale demonstrated good reliability with a Cronbach's α coefficient of 0.844 and KMO of 0.865. As part of the three factor CFA reported in Section 3.1, the behavioral intention items loaded significantly on their intended factor (standardized loadings ranged from 0.68 to 0.82, *p* < 0.001), supporting the construct validity of the scale in the current sample. This indicates that these items load on the same latent factor as the other intention focused items. The scale was retained in its original form to ensure comparability with prior studies. Nonetheless, it should be noted that the observed mediating effect of behavioral intention may partially reflect normative influences The present study retained all five original items (10): “I am willing to increase my opportunities for physical exercise,” “I am willing to follow the advice of significant others to participate in exercise,” “I am willing to follow a professional doctor's advice to participate in exercise,” “I can control my own exercise mode and volume,” and “If I decide to exercise, I will do so regardless of circumstances.” Responses were recorded on a 5-point Likert scale (1 = “Strongly Disagree,” 5 = “Strongly Agree”), with higher scores indicating stronger exercise behavior intention. In this study, the scale demonstrated good reliability and validity within the sample, with a Cronbach's α coefficient of 0.844 and a KMO was 0.865.

#### 2.2.3 International physical activity questionnaire short form

Physical activity levels were assessed using the International Physical Activity Questionnaire Short Form (IPAQ-SF), which includes seven items requiring participants to report the frequency and duration of their participation in activities of various intensities during the previous week (30). These activities include vigorous intensity activities (e.g., weightlifting, gym workouts, aerobics, or fast cycling), moderate intensity activities (e.g., carrying light loads, cycling at a regular pace, or brisk walking), walking, and sedentary behavior. The corresponding metabolic equivalent of task (MET) values were: walking, 3.3 METs; moderate intensity activities, 4.0 METs; and vigorous intensity activities, 8.0 METs. For data processing, truncation rules were applied: if the reported daily duration for any activity exceeded 3 h, it was capped at 180 min to ensure the total weekly duration for each activity level did not exceed 21 h (1,260 min). Following these criteria, 13 participants were excluded (nine due to extreme MET values, four due to incomplete data), resulting in a final analytical sample of 992. Physical activity levels were calculated in MET-minutes per week using the following formula: (walking minutes/day × walking days/week × 3.3) + (moderate-intensity minutes/day × moderate-intensity days/week × 4.0) + (vigorous-intensity minutes/day × vigorous-intensity days/week × 8.0).

### 2.3 Statistical analysis

A simple mediation model was tested using structural equation modeling (SEM) with observed variables in AMOS 26.0 (IBM, Armonk, NY, USA) to examine the proposed pathway from exercise self-efficacy to physical activity level through exercise behavioral intention. All constructs were treated as manifest variables using total scores (sum of exercise self-efficacy items, sum of exercise behavioral intention items, and IPAQ-SF MET-minutes per week). Demographic covariates, including gender, age, and education level, were included by regressing them onto both the mediator and the outcome. Maximum likelihood estimation was employed to estimate model parameters. The significance of direct and indirect effects was assessed using 5,000 bootstrap resamples with bias corrected 95% confidence intervals, with effects considered significant if the confidence interval did not include zero. Standardized regression coefficients (β) were reported for all paths, and total, direct, and indirect effects were used to calculate the proportion of the effect mediated. As the model was just identified (saturated), conventional fit indices were not applicable. Demographic covariates (gender, age, and education level) were retained in all models regardless of their statistical significance. This decision was made on *a priori* theoretical grounds, as these factors have been consistently associated with physical activity in prior research, and to control for potential confounding regardless of *p*-value.

## 3 Results

### 3.1 Assessment of common method bias

Procedurally, the questionnaire was administered anonymously, items were organized into separate sections, and participants were informed that there were no right or wrong answers. Statistically, Harman's single factor test was first conducted by entering all items from the three scales into an exploratory factor analysis. The unrotated solution extracted four factors with eigenvalues greater than 1, and the first factor accounted for 22.24% of the total variance, below the conventional 40% threshold (31), suggesting that CMB is unlikely to be a major concern.

A more rigorous examination using confirmatory factor analysis (CFA) showed that a single factor model with all 17 items loaded onto one latent method factor fit poorly [$\chi_{\left(119\right)}^{2}$ = 2345.8, CFI = 0.612, TLI = 0.578, RMSEA = 0.152, SRMR = 0.121], whereas the hypothesized three factor model (exercise self-efficacy, behavioral intention, and physical activity) fit acceptably [$\chi_{\left(116\right)}^{2}$ = 412.3, CFI = 0.951, TLI = 0.943, RMSEA = 0.056, SRMR = 0.045]. The large deterioration in fit (ΔCFI = 0.339, ΔRMSEA = 0.096) indicates that a single method factor cannot account for the shared variance. These results suggest that common method bias does not meaningfully distort the observed associations.

### 3.2 Demographic statistics

The study sample consisted of 992 vocational education students from Shanghai. Of these, 495 (49.9%) were male and 497 (50.1%) were female. In terms of educational level, 324 (32.6%) were vocational high school students, 342 (34.4%) were junior college students, and 326 (33.0%) were vocational university students, ensuring a balanced representation across these levels. Students' majors were distributed across six categories, with proportions of 23.2%, 18.8%, 20.8%, 19.2%, 17.6%, and 0.5%. The distribution of participants' residential areas was also fairly balanced, as shown in Table 1.

**Table 1:** General information of study subjects.

Variable	Category	Number	Percentage (%)/mean ±SD
Education level	Vocational high school	324	32.6
	Junior college	342	34.4
	Vocational university	326	33
Gender	Male	495	49.9
	Female	497	50.1
Age	-	-	20.4 ± 2.3
Major category	Engineering	230	23.2
	Medicine	186	18.8
	Humanities	206	20.8
	Business	190	19.2
	Arts	175	17.6
	Others	5	0.5
Daily residential area	Urban districts	442	45
	Suburban districts	550	55

*n* = 992.

### 3.3 Results of correlation analysis

Pearson correlation analysis was performed to assess the relationships among exercise self-efficacy, behavioral intention toward physical exercise, and physical activity level. The analysis revealed significant positive correlations between exercise self-efficacy and both physical activity level (*r* = 0.322, *p* < 0.001) and behavioral intention (*r* = 0.612, *p* < 0.001). Additionally, a significant positive correlation was found between physical activity level and behavioral intention (*r* = 0.346, *p* < 0.001). These results are shown in Table 2. According to conventional benchmarks (small: *r* = 0.10; medium: *r* = 0.30; large: *r* = 0.50), the correlation between exercise self-efficacy and behavioral intention was large (*r* = 0.612), whereas the correlations of these two variables with physical activity were of medium magnitude (*r* = 0.322 and 0.346, respectively). In the full mediation model, the squared multiple correlation for physical activity (*R*^2^ = 0.152) corresponds to a Cohen's *f*^2^ of approximately 0.18, indicating a medium effect size for the joint prediction of physical activity.

**Table 2:** Correlation analysis between exercise self-efficacy, exercise behavior intention, and physical activity level.

Variables	Physical activity level	Exercise self-efficacy	Exercise behavioral intention
Physical activity level	1	0.322^*^	0.346^*^
Exercise self-efficacy	0.322^*^	1	0.612^*^
Exercise behavioral intention	0.346^*^	0.612^*^	1

^*^P < 0.001; all variables were standardized, *n* = 992.

### 3.4 Mediating role of exercise behavioral intention

Exercise behavioral intention was examined as a mediator in the relationship between exercise self-efficacy and physical activity level using path analysis in AMOS. Results indicated that exercise self-efficacy was a significant positive predictor of exercise behavioral intention (β = 0.609, *p* < 0.001). When both exercise self-efficacy and exercise behavioral intention were included as predictors of physical activity level, each variable remained a significant positive predictor (β = 0.148 for self-efficacy, *p* < 0.001; β = 0.201 for behavioral intention, *p* < 0.001; see Table 3).

**Table 3:** Standardized regression coefficients from AMOS path analysis (*n* = 992).

Variables	Exercise behavior intention (Model 1)	Physical activity levels (Model 2)
	β	β
Exercise self-efficacy	0.609^*^	0.148^*^
Exercise behavior intention	—	0.201^*^
Education level	0.045^*^	0.047^*^
Gender	0.026	0.006
Age	−0.001	−0.002
*R* ^2^	0.376	0.151
Adjusted *R*^2^	0.373	0.145

^*^P < 0.001; In Model 1, the dependent variable was exercise intention, with exercise self-efficacy and demographic control variables as predictors. In Model 2, the dependent variable was physical activity level, with exercise self-efficacy and demographic control variables as predictors. All variables were standardized by *Z*-score before being entered into the regression equations; “—” indicates that the variable was not included in the corresponding regression model; β values are retained to three decimal places; *R*^2^ and adjusted *R*^2^ are retained to three decimal places.

Mediation analysis based on 5,000 bootstrap resamples revealed a significant direct effect of exercise self-efficacy on physical activity level (β = 0.148, 95% CI: 0.084 to 0.211, *p* < 0.001). The indirect effect through exercise behavioral intention was also significant (β = 0.122, 95% CI: 0.085 to 0.163, *p* < 0.001), indicating partial mediation. The direct and indirect effects accounted for 54.81% and 45.19% of the total effect, respectively (Table 4).

**Table 4:** Mediation analysis results from AMOS (*n* = 992).

Effect type	Effect size	SE	95% CI		Relative mediation effect (%)
			LCL	UCL	
Total effect	0.270^*^	0.026	0.219	0.319	-
Direct effect	0.148^*^	0.033	0.084	0.211	54.81
Mediated effect	0.122^*^	0.020	0.085	0.163	45.19

All core variables in the model were standardized using *Z*-score transformation.^*^P < 0.001; Bootstrap = 5,000, 95% CI.

## 4 Discussion

### 4.1 Theoretical implications of main findings

**Figure 1:**
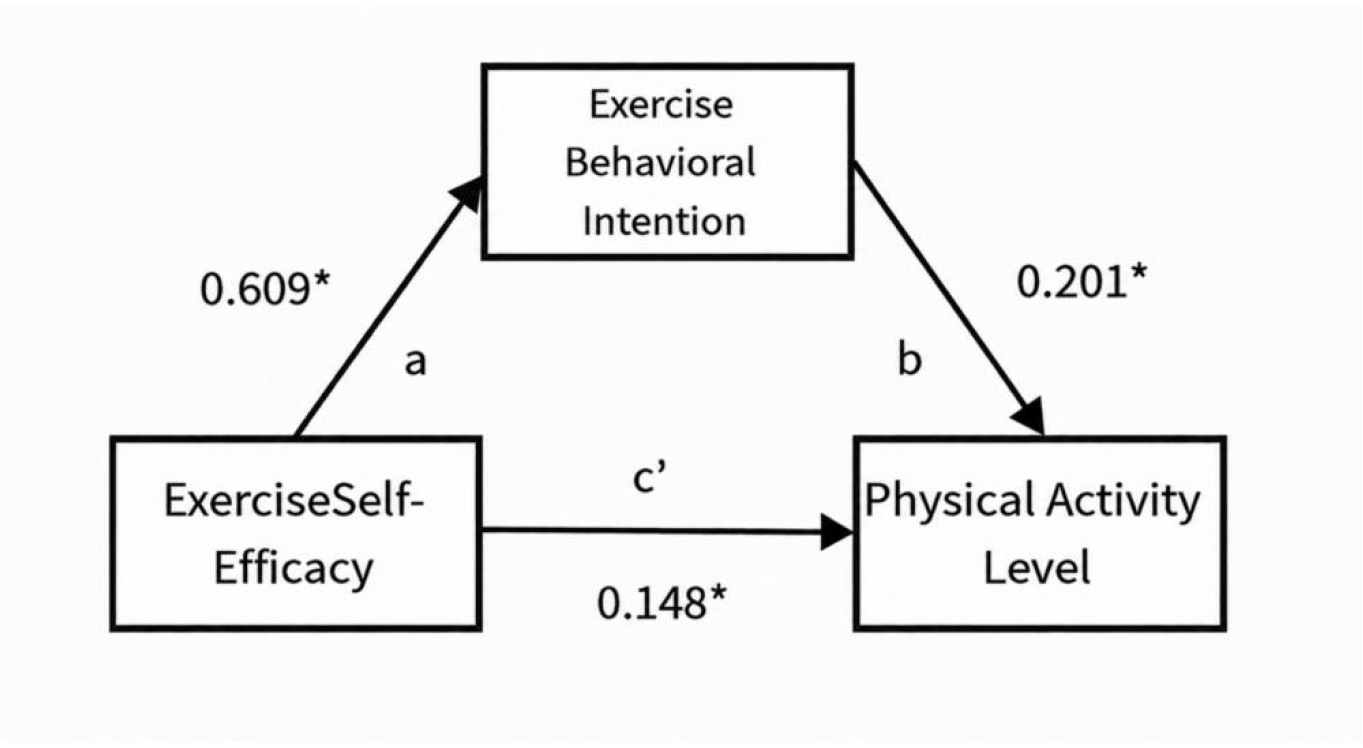
Mediation model with standardized coefficients from AMOS. *a* = 0.609, *b* = 0.201, *c*′ = 0.148. *p < 0.001.

This study confirms that exercise behavioral intention partially mediates the relationship between exercise self-efficacy and physical activity levels among vocational education students in Shanghai, with the mediating effect accounting for 45.19% of the total variance. The mediation proportion of 45.19% indicates that nearly half of the effect of exercise self-efficacy on physical activity operates through behavioral intention. In behavioral intervention research, mediated effects of around 30% are often considered practically meaningful (7), suggesting that intention formation constitutes a central pathway among vocational students rather than a minor one. Nonetheless, the remaining 54.81% direct effect indicates that self-efficacy also influences physical activity via other unmeasured pathways, such as automatic behavioral regulation or environmental facilitation. This finding contributes to the theoretical integration of Bandura's Social Cognitive Theory and Ajzen's Theory of Planned Behavior within a previously underexplored population (22). Specifically, the results empirically support the process in which efficacy beliefs contribute to the formation of exercise intentions, which in turn are associated with the execution of physical activity. This distinction is particularly salient for vocational education students, whose academic and daily schedules are predominantly oriented toward professional skill acquisition, often at the expense of physical activity. The theoretical pathway illustrated in Figure 1 describes the hypothesized mediation mechanism of this study. Exercise self-efficacy not only directly predicts physical activity but also indirectly influences it by enhancing exercise behavioral intention. This mediation mechanism extends current knowledge by showing that behavioral intention functions as a key cognitive bridge linking confidence to action even among populations facing substantial external constraints. The demanding academic and professional training schedules characteristic of vocational education may contribute to lower levels of physical activity. Accordingly, interventions aimed at enhancing the pathway from self-efficacy to intention and ultimately to behavior are especially important for this population.

Theoretically, our findings bridge Social Cognitive Theory and the Theory of Planned Behavior by specifying a mechanism in which self-efficacy influences physical activity partly through behavioral intention. However, this integration is not without tension. TPB conceptualizes perceived behavioral control as a direct predictor of both intention and behavior, which conceptually overlaps with self-efficacy. In our model, self-efficacy was operationalized as task specific confidence, yet PBC was not measured separately, leaving it unclear whether self-efficacy exerts additional indirect effects beyond PBC. Future research should use confirmatory factor analysis to disentangle these constructs in vocational student samples.

### 4.2 Comparison with previous research

The direct effect of self-efficacy on physical activity in our vocational student sample (β = 0.148) was lower than the direct effect of self-efficacy on behavior reported by Hong and Chung (10) among general university students (β = 0.179 in their main model). This discrepancy may reflect differences in populations and theoretical pathways. First, vocational students face greater structural barriers (e.g., mandatory internships, shift schedules) that hinder the direct translation of confidence into action. Second, prior university samples typically had higher baseline physical activity levels, potentially inflating the self-efficacy–behavior association. Third, our study included rigorous covariate adjustments (gender, age, education level), whereas some previous studies did not control for these confounders.

Importantly, the mediating proportion in our study (45.19%) exceeds the 30%−40% range often reported in college student samples, suggesting that the self-efficacy → intention → behavior pathway may not be uniform across educational contexts. Specifically, when external supports are limited, as is common in vocational settings, individuals may rely more heavily on conscious intentions to bridge the association between believing and doing. This interpretation aligns with Hou et al. (11), who found that self-efficacy moderates the intention behavior gap primarily under high barrier conditions. Future research should examine whether the mediating effect is moderated by contextual factors such as campus sports culture or internship intensity.

The heightened mediating role of intention in vocational students can be understood in light of their unique contextual characteristics. As noted in the introduction, recent evidence from Shanghai vocational schools indicates a smartphone addiction prevalence of 70.5% and musculoskeletal disorder prevalence of 38.0% (21). Such conditions may directly undermine self-efficacy (e.g., “I cannot exercise because of back pain”) and disrupt automatic behavioral routines, increasing reliance on deliberate intentions. Additionally, vocational curricula often include 6–8 months of off campus internships, during which campus sports facilities are largely inaccessible (19). Under these fragmented schedules, forming a strong behavioral intention becomes a prerequisite rather than a supplement for engaging in physical activity. Consequently, the intention-mediated pathway is not merely present but functionally essential in this population.

### 4.3 Practical implications and intervention strategies

Based on the effect sizes and sample characteristics observed in this study, several evidence guided intervention strategies are recommended for vocational education students. First, given that behavioral intention mediates 45.19% of the total effect of self-efficacy on physical activity (indirect effect β = 0.122), interventions should prioritize intention formation alongside confidence building activities. Although the direct effect of self-efficacy remains substantial (β = 0.148), targeting intention ensures a more complete translation of confidence into behavior. Second, to address the intention behavior gap, interventions should consider context specific barriers, such as fragmented schedules, limited access to facilities for the 55% of students residing in suburban districts, high smartphone usage (70.5%), and musculoskeletal complaints (38.0%) (21). Strategies may include app, based walking challenges for students with high smartphone use and low impact or seated exercises for those with physical limitations. Third, interventions should be tailored to educational level and gender. Younger vocational high school students may benefit from structured, peer led sessions, while vocational university students may prefer flexible, self paced plans compatible with internship schedules. Single gender sessions could also be feasible, particularly to support female students who may report lower self-efficacy. Fourth, physical activity should be embedded into existing institutional routines to minimize additional time demands. Brief micro interventions lasting around 5 min, such as using stair only pathways, incorporating standing breaks during theory classes, or engaging in short activities during class transitions and internship shift changes, can promote student engagement without adding to their workload. This approach is particularly important given the intensive professional skill requirements of vocational curricula (19). Annual screening using the IPAQ-SF could identify students with insufficient activity (< 600 MET-minutes/week), who may then receive targeted interventions, with accelerometer based assessments for those with very low activity levels to personalize programs. Collectively, these strategies integrate psychological and environmental considerations to enhance the likelihood that intentions translate into actual physical activity.

### 4.4 Limitations

This study has several limitations. First, the cross sectional design limits the ability to draw causal inferences. Second, physical activity was assessed via self report, which may be subject to social desirability bias. Third, the sample was restricted to vocational institutions in Shanghai, potentially limiting the generalizability of the findings to other regions or less rich resource settings. Fourth, the model included only a single mediator, this decision was guided by the Theory of Planned Behavior, which identifies behavioral intention as the most proximal antecedent of physical activity. The primary aim of the study was to examine this key pathway in an understudied population. leaving other potentially relevant variables, such as social support, planning, and environmental accessibility unexamined. Future research should expand to more diverse geographic regions and institutional types, and incorporate additional mediators to provide a more comprehensive understanding of the mechanisms linking self-efficacy to physical activity. Fifth, as noted in the methods Section 2.2.2, two items of the exercise behavioral intention scale conceptually overlap with the subjective norms component of the Theory of Planned Behavior. Although confirmatory factor analysis supported a unidimensional factor structure for the five items, this overlap may have inflated the observed association between behavioral intention and physical activity, because subjective norms can directly influence behavior independent of intention. Consequently, the mediating effect attributed to behavioral intention might partly reflect normative influences rather than pure motivational intention. Future research should incorporate these constructs as parallel or sequential mediators to provide a more comprehensive understanding of the mechanisms linking self-efficacy to physical activity in vocational students. Future research should also expand to more diverse geographic regions and institutional types.

## 5 Conclusion

Exercise self-efficacy is associated with physical activity both directly and indirectly via behavioral intention in vocational students. Interventions enhancing self-efficacy and behavioral intention may support higher activity levels, though longitudinal studies are needed to confirm directionality.

## Data Availability

The raw data supporting the conclusions of this article will be made available by the authors, without undue reservation.
